# Ultrasensitive RNase H activity detection using the transcription-based hybrid probe and CRISPR/cas12a signal amplifier

**DOI:** 10.3389/fphar.2025.1589150

**Published:** 2025-04-11

**Authors:** Sheng Ding, Yinghua Wei, Minglong Yang, Jinyi Shi, Kaiyuan Ren, Xinli Li, Zhuo Tang

**Affiliations:** ^1^ Clinical Medical College and Affiliated Hospital, Chengdu University, Chengdu, China; ^2^ Guangzhou National Laboratory, Guangzhou, China; ^3^ Natural Products Research Center, Chengdu Institute of Biology, Chinese Academy of Sciences, Chengdu, China

**Keywords:** Rnase H, CRISPR/Cas12a, activity detection, inhibitor screening, hybprobe

## Abstract

Ribonuclease H (RNase H), a critical functional protein in replication and genome stability, is emerging as a crucial therapeutic target for various diseases, including immune disorders. We present a transcription-based hybrid probe, referred to as Hybprobe, and a CRISPR/Cas12a signal amplifier for the rapid, sensitive, and low-cost detection of RNase H activity. In this method, the RNA strand of the Hybprobe is specifically cleaved by RNase H, releasing a single-stranded DNA activator that facilitates recognition and cleavage by the Cas12a/crRNA complex, triggering signal amplification via Cas12a′s trans-cleavage activity. The proposed method demonstrates ultra-high sensitivity, capable of detecting RNase H as low as 9.02 × 10^−10^ U/μL, making it approximately 1,000 times more sensitive than several previously reported methods. Furthermore, we demonstrated the application of this method for RNase H inhibitor evaluation and its practical use across various biological samples, including cell extracts and HIV reverse transcriptase. In summary, the results suggest that this method is a promising tool for the highly sensitive detection of RNase H and the diagnosis of diseases associated with RNase H.

## Graphical Abstract

**Figure:**
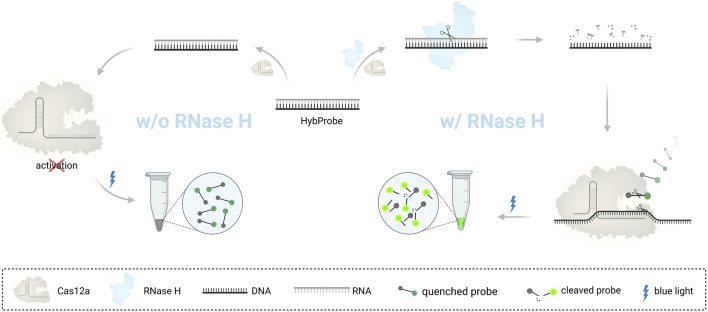

## Introduction

RNase H (Ribonuclease H) is a highly conserved enzyme that specifically hydrolyzes the RNA strand in DNA-RNA hybrid duplexes (Nowotny et al., 2005). It plays crucial roles in various biological processes, including DNA replication, repair, transcriptional regulation, and antiviral defense mechanisms (Cerritelli and Crouch, 2009). For instance, RNase H is essential for removing RNA primers during DNA synthesis, ensuring the integrity of newly synthesized DNA strands (Bubeck et al., 2011). It also regulates gene expression by modulating RNA-DNA hybrid structures (Arraiano et al., 2010) and participates in the immune response against viral infections by degrading viral RNA-DNA intermediates (Moelling et al., 2017). Aberrant RNase H activity has been implicated in several disorders. In cancer, altered RNase H levels may disrupt genomic stability and DNA repair mechanisms, contributing to tumor progression (Feng and Cao, 2016; Aden et al., 2019). In immune system, RNase H deficiency is associated with some autoimmune diseases such as Aicardi-Goutières syndrome (AGS) (Coffin et al., 2011; Bartsch et al., 2017). Additionally, RNase H is a critical component of the HIV-1 reverse transcriptase complex, making it a potential target for antiretroviral therapy (Klumpp et al., 2003; Kang et al., 2023). Overall, RNase H is vital for fundamental cellular processes and being a potentially therapeutic target for associated diseases. Thus, developing sensitive and specific methods for RNase H activity assay has been drawing increasing attention.

Traditional methods for RNase H activity detection are mainly based on polyacrylamide gel electrophoresis (PAGE) (Chan et al., 2004), high-performance liquid chromatography (HPLC) (Frank et al., 1999), and electrochemical biosensors (Tian et al., 2022). These methods, while effective, are often time-consuming and labor-intensive, requiring sophisticated instrumentation and multiple-step separation procedures. For instance, PAGE and HPLC methods typically involve the use of radio-labeled substrates and electrophoretic separation, which are not suitable for large-scale sample analysis due to their complexity and high cost. Additionally, the detection sensitivity of these techniques is usually insufficient to detect trace amount of RNase H, which can limit their practical application in high-throughput inhibitor screening and cellular RNase H activity monitoring. To overcome these challenges, some state-of-the-arts strategies have been proposed. Lee et al. developed a fluorescent dye-based RNase H assay by coupling with rolling circle amplification (RCA), which could detect as low as 0.019 U/mL RNase H within 5 min (Lee et al., 2017b). Apart from the use of isothermal amplification method to enhance the sensitivity, other signal amplification methods also have been reported, such as phosphorothioated-terminal hairpin formation and self-priming extension (PS-THSP) (Yoon et al., 2024), catalytic hairpin assembly (CHA) (Lee et al., 2017a), exponential amplification reaction (EXPAR) (Zhang et al., 2024). Additionally, some groups also reported some easy but efficient strategies by integrating functional nucleic acids or nanomaterials (Zhao et al., 2017; Wang et al., 2020; Hu et al., 2021; Zhang et al., 2021). Although these methods presented some progress in cost and simplicity, they still require long incubation time, step-wise operations or lack adequate sensitivity. Therefore, developing a new method with high sensitivity, easy-to-use operation and low cost is still desirable.

In recent years, the CRISPR/Cas system, particularly Cas12a (also known as Cpf1), has been widely applied in gene editing, molecular diagnostics, and biosensing (Wu et al., 2020; Tang et al., 2021). Cas12a is an RNA-guided DNA endonuclease DNA cleavage activity. Upon recognizing a specific DNA sequence, Cas12a exerts single-strand DNase activity that nonspecifically cleave the ssDNA (Li et al., 2018). This feature makes Cas12a a powerful tool for signal amplification, capable of amplifying detection signal, thereby greatly enhancing detection sensitivity. Owing to its high specificity and programmability, Cas12a has been widely used as signal amplifier to detect diverse targets including nucleic acids, proteins, and small molecules (Li et al., 2020; Shi et al., 2021). Most recently, Xie et al. reported a method using Cas12a coupled with primer exchange reaction (PER) to detect RNase H (Xie et al., 2022). While it provided a sensitive alternative for RNase H assay, the complicated PER design and long testing time may hinder its broad use. In our study, we herein developed a simple and one-pot method by using DNA/RNA hybrid probe and Cas12a amplifier to realize ultrasensitive detection of RNase H. To be noted, the hybrid probe used in our study was easily prepared, which made our method even simpler and cost-effective. In addition, the feasibility of inhibition assay was also investigated using this method.

## Materials and methods

### Materials

Cas12a (Cpf1) was purchased from Kxbiotech Co., Ltd. (Beijing, China); 10× NEB buffer 1 (B7001S), 10× NEB buffer 2 (B7002S), 10× NEB buffer 4 (B7004S), and apurinic/apyrimidinic endonuclease 1 (APE1) were purchased from New England Biolabs (NEB); uracil-N-glycosylase (UNG), gentamicin, NTPs, and dNTPs were purchased from Sangon Biotechnology Co., Ltd. (Shanghai, China); T4 polynucleotide kinase (PNK), T7 RNA polymerase, and 5× Transcription buffer were purchased from Thermo Fisher Scientific, USA; RIPA cell lysis buffer and BCA protein concentration assay kit were purchased from New Cell and Molecular Biotech Co.,Ltd. (Suzhou, China); RNase H and 10× EasyTaq buffer were purchased from TransGen Biotech Co., Ltd. (Beijing, China); Thermostable RNase H and RNase A were purchased from Beyotime Biotechnology Co., Ltd. (Shanghia, Chian); HIV-p66 recombinant protein was purchased from Sino Biological, Inc. (China). All oligonucleotide sequences (shown in Supplementary Table S1) were synthesized by Sangon Biotechnology Co., Ltd. The quality and concentration of oligonucleotide sequences were determined by the ultra-micro ultraviolet spectrophotometer ND5000 (BioTeke Co., Ltd., Beijing).

### Generation of hybrid probe (HybProbe)

0.2 µM HybProbe template was mixed with 0.2 µM T7 promoter sequence (T7F) in 1× EasyTaq buffer and undergone high-temperature denaturation, followed by slow annealing. The program was set as follows: 95°C for 5 mins; cooling at 0.1°C/s to 37°C. Then, proceed with the transcription of the HybProbe. The total reaction volume was 50 μL, containing 1× Transcription buffer, 100 U of T7 RNA polymerase, 0.5 mM NTPs, 15 µL of the annealed product of HybProbe template and T7F. The reaction was incubated at 37°C for 15 mins, followed by a 75°C treatment for 10 min to denature the RNA polymerase. After cooling to room temperature, the HybProbe and generated crRNA were roughly purified by ethanol precipitation and quantified by ultraviolet spectrophotometer ND5000.

### One-step RNase H detection

A total volume of 10 μL RNase H detection reaction consisted of 1× NEBuffer2, 40 nM Cas12a, 0.625 μM single-stranded signal reporter sequence (ssDNA Reporter), 30 nM HybProbe and crRNA complex, and RNase H at specific amount. The reaction was conducted in a real-time fluorescence PCR instrument at 37°C for 20 mins, with fluorescence signals recorded at 1-min intervals. Unless otherwise specified, all error bars in the results represent data obtained from three independent experimental replicates.

### Sensitivity and specificity assay

For sensitivity assay, serially diluted RNase H were added to the above reaction. To calculate the limit of detection (LOD), the 3σ rule was applied. Due to the linear relationship between the logarithm of concentration and the signal, equation Y = AVE (blank) + 3SD (blank) from the obtained linear regression model is utilized. The corresponding value is then derived by taking the inverse logarithm and was considered as an approximation of the LOD. For specificity assay, RNase H (0.05 U/μL), thermostable RNase H (0.05 U/μL), RNase A (100 μg/mL), PNK(1 U/μL), BSA (1 mg/mL), and APE1 (1 U/μL) were added to the detection reaction.

### Inhibition assay

The RNase H inhibition assay was preformed using the model inhibitor gentamicin. Different concentrations of gentamicin were first incubated with RNase H at a final concentration of 1 × 10^−5^ U/μL in 1× NEBuffer2. After incubation at 37°C for 10 mins, 40 nM Cas12a, 0.625 μM ssDNA Reporter, and 30 nM HybProbe-crRNA mix were added to the reaction mixture for fluorescence intensity measurement, following the detection procedure described above. To evaluate the inhibition efficiency, relative activity (RA) is calculated using the formula RA = (Fi - Fnc)/(Fpc - Fnc), where Fi represents the fluorescence intensity of the reaction with the inhibitor, Fpc denotes the fluorescence intensity of the reaction with water, and Fnc stands for the fluorescence intensity of the negative control.

### Preparation of cell extracts

Human breast cancer cell line MCF-7, human tissue cell lymphoma cell line U937, and human embryonic kidney cell line 293 T were cultured in DEME medium (containing 10% fetal bovine serum, 100 U/mL penicillin, and 100 mg/mL streptomycin) under 5% CO_2_ at 37°C. When the cell count reached approximately 500,000, cells were collected and treated with RIPA cell lysis buffer on ice for 30 min. Then, the cells were centrifuged at 12,000 rpm for 10 mins, and the supernatant was stored at −20°C for future use. When needed, protein was diluted using the lysis buffer.

## Results

### The principle of RNase H detection based on HybProbe and Cas12a

**FIGURE 1:**
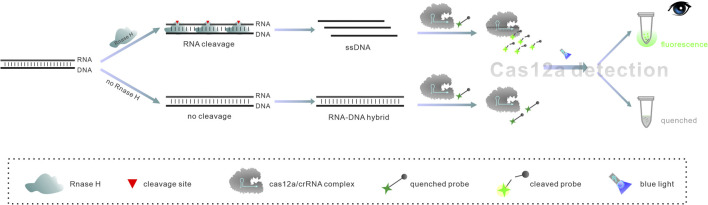
Schematic diagram of the RNase H detection based on HybProbe.

Based on the specific function of RNase H to recognize and digest RNA in DNA-RNA hybrid strands, we developed a one-step detection strategy for RNase H using the CRISPR/Cas12a system. As illustrated in Figure 1, we designed a hybrid probe (HybProbe), in which the target DNA sequence is blocked by the RNA strand. It was worth mentioning that the RNA strand was intentionally designed as the guide RNA (crRNA) of Cas12a, thus the HybProbe and crRNA could be easily prepared by *in vitro* transcription (IVT). In the presence of RNase H, the RNA strand of the HybProbe was cleaved, thereby releasing the target ssDNA sequence. Meanwhile, the pre-added Cas12a could bind with the extra crRNA produced by IVT and form a riboprotein complex that could specifically recognize the released ssDNA and activate the trans-cleavage activity of Cas12a. This resulted in the cleavage of a single-stranded signal reporter (ssReporter) sequence labeled with a fluorophore and a quencher group, generating a fluorescence signal. In contrast, in the absence of RNase H, the ssDNA activator was blocked within the HybProbe, preventing the Cas12a/crRNA binary complex from activating the collateral cleavage of ssReporter. As a result, no fluorescence signal could be produced.

### The feasibility of the strategy

**FIGURE 2:**
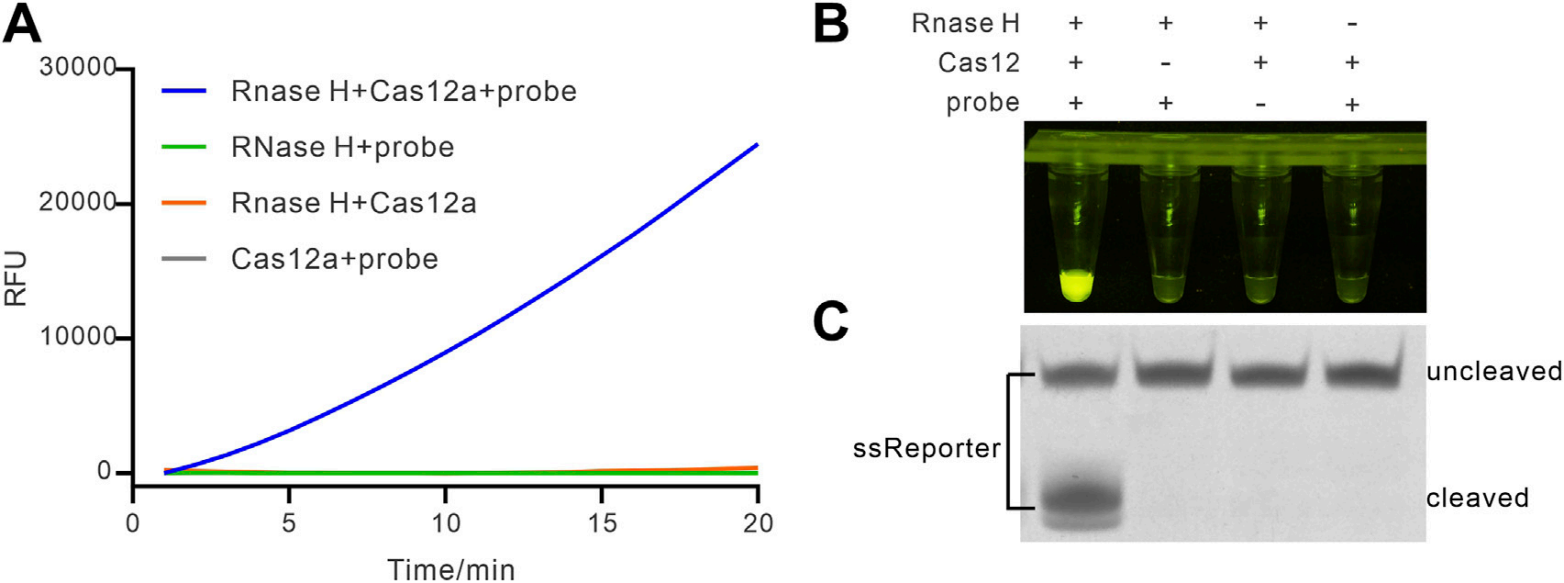
Feasibility of HybProbe for APE1 detection. **(A)** Real-time fluorescence assay of CRISPR/Cas12a signal amplifier triggered by different reactions containing different components. **(B)** End-point fluorescence intensity difference between the reactions with different components. **(C)** Electrophoretic analysis of cleavage of ssReporter.

To examine the feasibility of the method, we first simulated the HybProbe by artificially hybridizing synthetic crRNA and ssDNA in a ratio of 5 to 1. Then we conducted the feasibility assay by investigating the impact of different components on the reaction. As can be seen in Figure 2A, the rising fluorescence curve could only be present when all the components including RNase H, Cas12a, and HybProbe were added simultaneously. While lacking any part of these reagents, no significant fluorescence signals could be detected. Additionally, the endpoint visualized image under the blue light (λ = 475 nm) demonstrated that an obvious fluorescence could only be observed when RNase H and the other components were present together (Figure 2B). Furthermore, we evaluated the cleavage of the quenched ssDNA reporter by denatured polyacrylamide gel electrophoresis (PAGE). As displayed in Figure 2C, the cleaved band of ssReporter could be clearly found in the reaction containing RNase H and other components, which was consistent to the real-time fluorescence and endpoint visualization results. Taken together, these results demonstrated that the HybProbe could be used for the detection of RNase H.

### Optimization of the transcription-based HybProbe and detection condition

**FIGURE 3:**
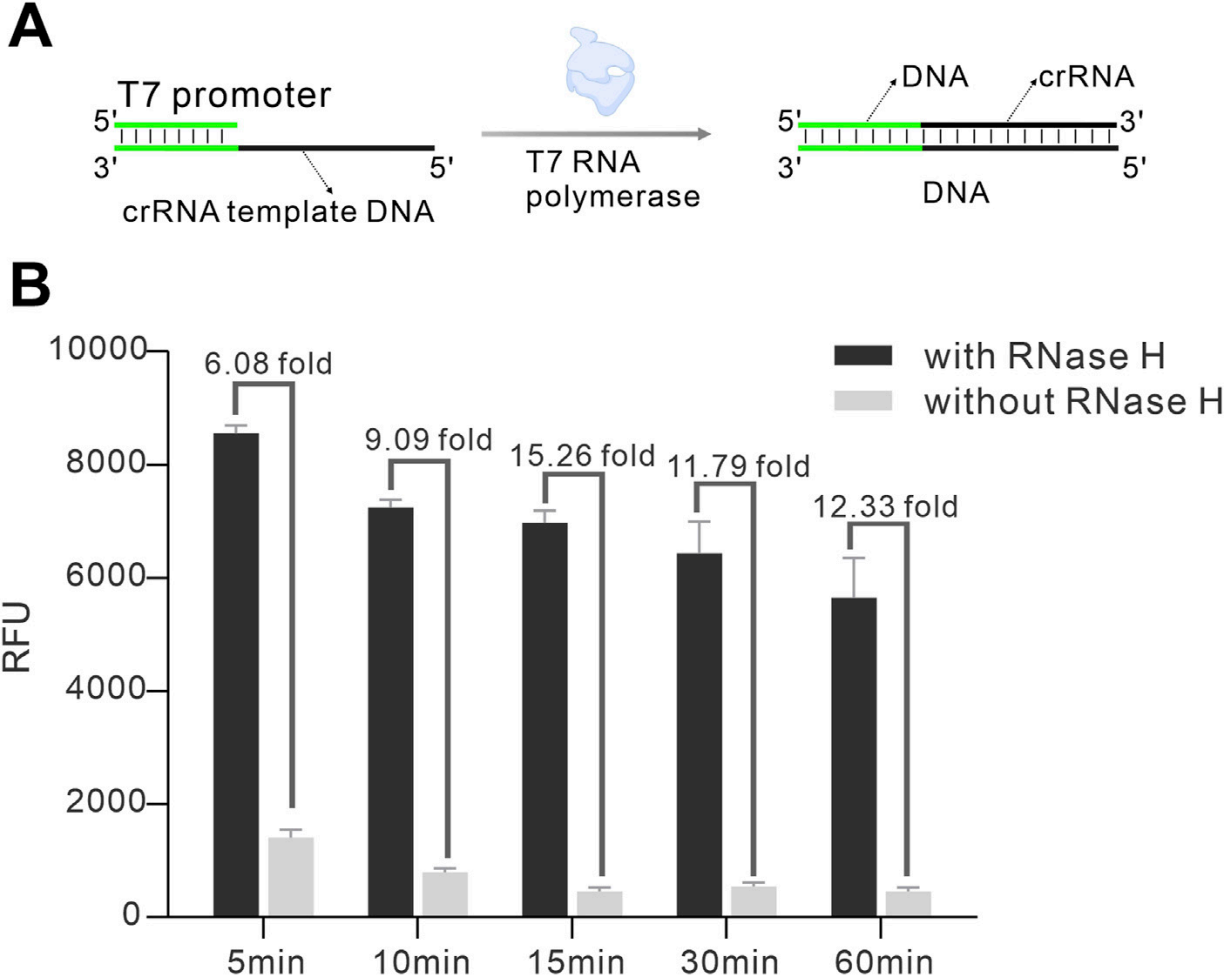
Optimization of the time for Hybprobe preparation and crRNA transcription. **(A)** Schematic of HybProbe preparation and crRNA transcription. **(B)** Investigation of the effect of different transcription times on the detection signal.

It has been reported that T7 RNA polymerase was capable of transcribing single-stranded DNA templates containing a double-stranded promoter region. In this way, the RNA transcribed by such template was complementary to the ssDNA region of the template. Based on this phenomenon, we hypothesized that crRNA generated from a single-stranded template could act as a blocking sequence to form a HybProbe. Thus, both the crRNA and HybProbe could be produced *in situ*, making it cost-effective and easy-to-use. Encouraged by that, in this study, we used *in vitro* transcription (IVT) to simultaneously obtain hybridization probes and crRNA. As shown in Figure 3A, a single-stranded DNA transcription template with a crRNA complementary sequence (black region of the template strand) and a T7 promoter sequence (green region of the template strand) forms a partial double-stranded structure. When introduced to IVT, a significant amount of crRNA was transcribed by T7 RNA polymerase. Simultaneously, a DNA-RNA hybrid probe (HybProbe) that contained partial double-stranded DNA was formed as some of the crRNA acted as the blocking sequence of template DNA. In other words, a portion of the transcribed crRNA was used to block the template DNA, while the remainder acted as the guide RNA to bind with Cas12a. As the crRNA and HybProbe was the key to develop the method, we sought to optimize the transcription time. As illustrated in Figure 3B, while 5 min of transcription allowed the transcription product to be used for RNase H detection, it still produced background noise, indicating that produced crRNA was insufficient to block the template. As the transcription time increased, the RNase H-induced fluorescence signal gradually decreased; however, the background signal also diminished, which likely due to excess crRNA produced to compete with the Cas12a/crRNA complex for binding to the DNA template. Finally, 15 min was selected as the optimal transcription time for crRNA production and HybProbe preparation, as it yielded the highest signal-to-noise ratio. Compared to other RNase H detection methods using Cas12a, this strategy required only a short transcription period to obtain both HybProbe and crRNA, offering significant advantages in reducing detection time and costs. Next, we optimized the detection condition including reaction buffer, Cas12a concentration and reporting temperature. The results reflected that NEB buffer 2, 40 nM of Cas12a, and 37°C were the optimal condition for RNase H detection (Supplementary Figures S1, S2), which were selected for the following experiments.

### Analytical performance of RNase H detection

**FIGURE 4:**
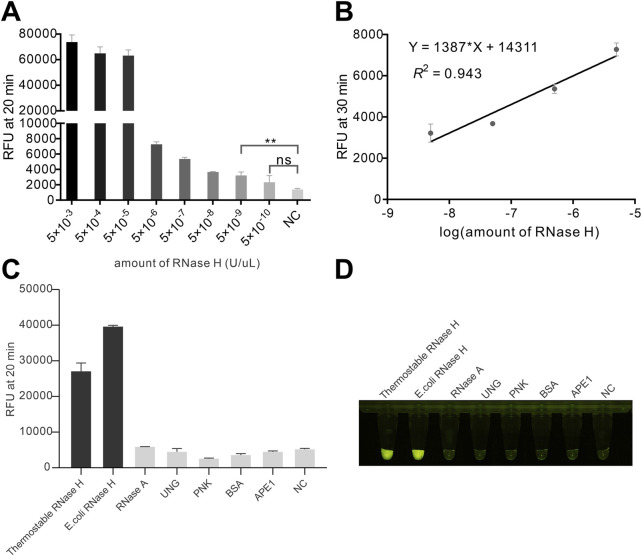
Investigation of the sensitivity and the specificity of the assay. **(A)** Endpoint fluorescence intensity at different RNase H concentrations. **(B)** The linear relationship between fluorescence intensity and the logarithm of RNase H concentration. **(p < 0.01) indicates a significant difference; ns indicates no significant difference (p > 0.05). **(C)** Detection of endpoint fluorescence intensity for different proteins. **(D)** Endpoint visualization results from Figure **(C)**. The concentrations of thermostable RNase H, RNase H, RNase A, PNK, BSA, and APE1 used were 0.05 U/μL, 0.05 U/μL, 1 U, 100 μg/mL, 10 U, 1 mg/mL, and 10 U, respectively.

After the optimization of the reaction condition, we intended to investigate the analytical performance of the method. By adding serially diluted RNase H, we aimed to determine the sensitivity of the method. We observed a progressive decrease in fluorescence signal intensity as the RNase H concentration decreased. Notably, when the RNase H concentration was below 5 × 10^−5^ U/μL, the fluorescence signal intensity decreased sharply (Figure 4A; Supplementary Figure S3). Besides, there was a strong linear relationship between the fluorescence intensity and the logarithm of the RNase H concentration ranging from 5 × 10^−5^ to 5 × 10^−9^ U/μL (Figure 4B). The limit of detection (LOD) of was estimated to be 9.02 × 10^−10^ U/μL, which was about 1,000 times more sensitive than many reported methods (Supplementary Table S2). To investigate the specificity of the proposed method, we compared RNase H from two different sources with several functional proteins involved in nucleic acid metabolism. As shown in Figure 4C, both RNase H from different sources induced strong fluorescence signals, while the fluorescence signal intensities generated by non-target proteins including RNase A, UNG, PNK, and APE1, were comparable to negative control. Additionally, the endpoint fluorescence induced by RNase H could be easily distinguished from the other control groups (Figure 4D). These results suggested that the developed strategy offered high specificity and sensitivity for RNase H activity detection. This high sensitivity was likely due to the strong signal amplification ability of the Cas12a and the rational design of HybProbe. Besides, the whole detection could be finished within 45 min, leading this method an appealing choice in practical application. In addition, the high simplicity and low cost (Supplementary Table S3) of the method makes it adaptable to realize high-throughput inhibitor screening, which is attractive in drug development.

### RNase H inhibition assay

**FIGURE 5:**
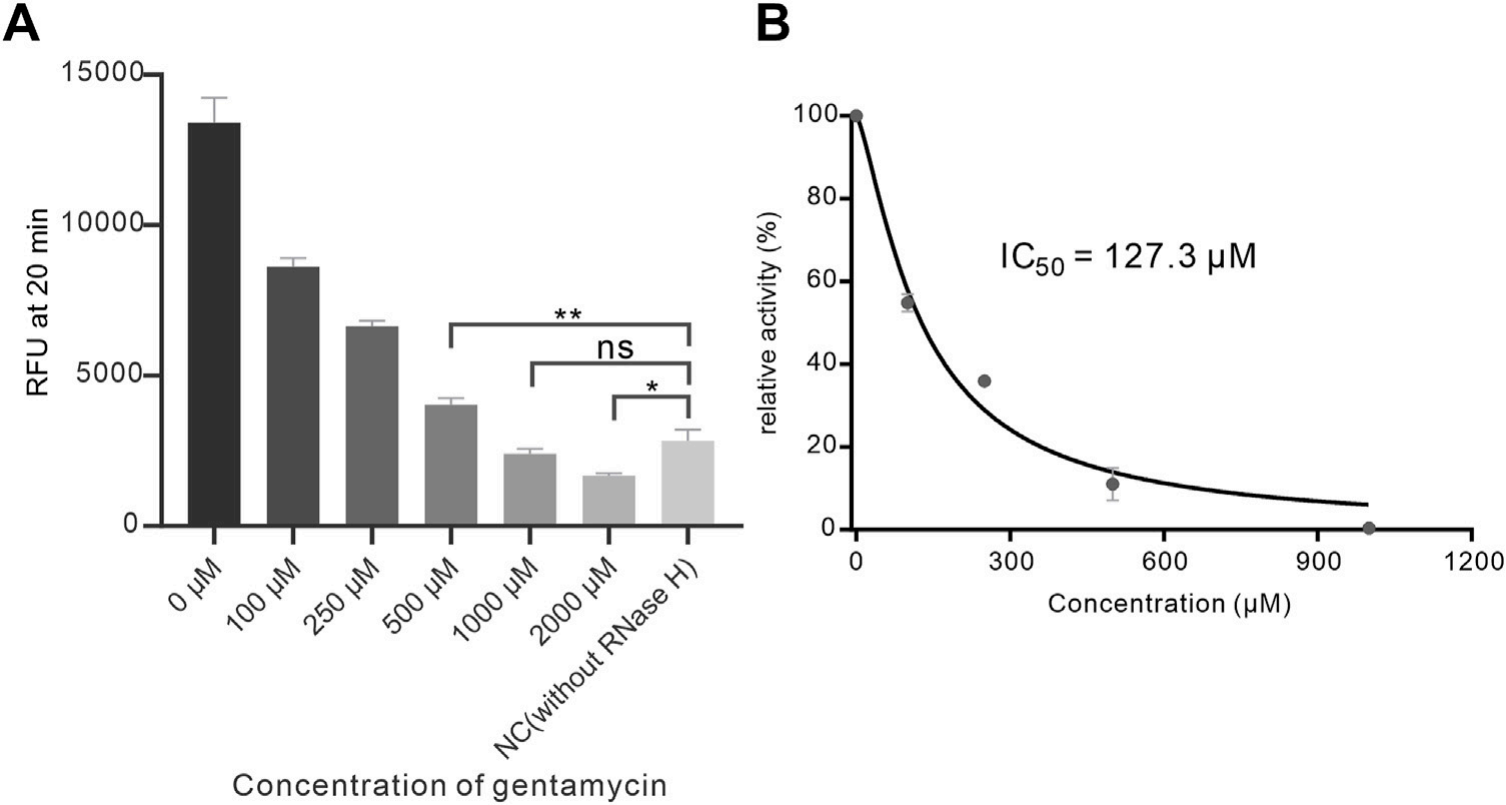
Inhibition assay of RNase H. **(A)** Investigation of the inhibition of RNase H (final concentration 1 × 10^-5 U/μL) by different concentrations of gentamicin; **(B)** Analysis of the inhibition efficiency of gentamicin. * (p < 0.05) and ** (p < 0.01) indicate significant differences; ns indicates no significant difference (p > 0.05).

Since RNase H plays a critical role in many physiological or pathological processes, it has been recognized as a potential therapeutic target for many disorders including viral infection and tumor. Thus, we intended to perform RNase H inhibition assay to evaluate the method. Herein, the reported inhibitor gentamicin was chosen to inhibit RNase H (Song et al., 2014). We pre-incubated varying concentrations of gentamicin with a fixed concentration of RNase H for 5 min before adding them to the detection system. As shown in Figure 5A and Supplementary Figure S4, increasing concentrations of gentamicin led to a gradual decrease in fluorescence intensity, indicating a concentration-dependent inhibition of RNase H activity by gentamicin. Interestingly, we observed that the fluorescence intensity from the inhibition reaction with 2 mM gentamicin was even lower than that of the negative control (without RNase H), which could be explained by that high concentration of gentamicin might inhibit Cas12a activity. Furthermore, the half-maximal inhibitory concentration was (IC_50_) of gentamicin for RNase H was estimated to be 127.3 μM (Figure 5B). Apart from the gentamicin, we also successfully validated the inhibition assay using another inhibitor streptomycin with a IC_50_ at 106 μM (Supplementary Figure S5). These results suggested that our method was promise for RNase H inhibition analysis and inhibitor screening.

### Detection of RNase H from different organisms

**FIGURE 6:**
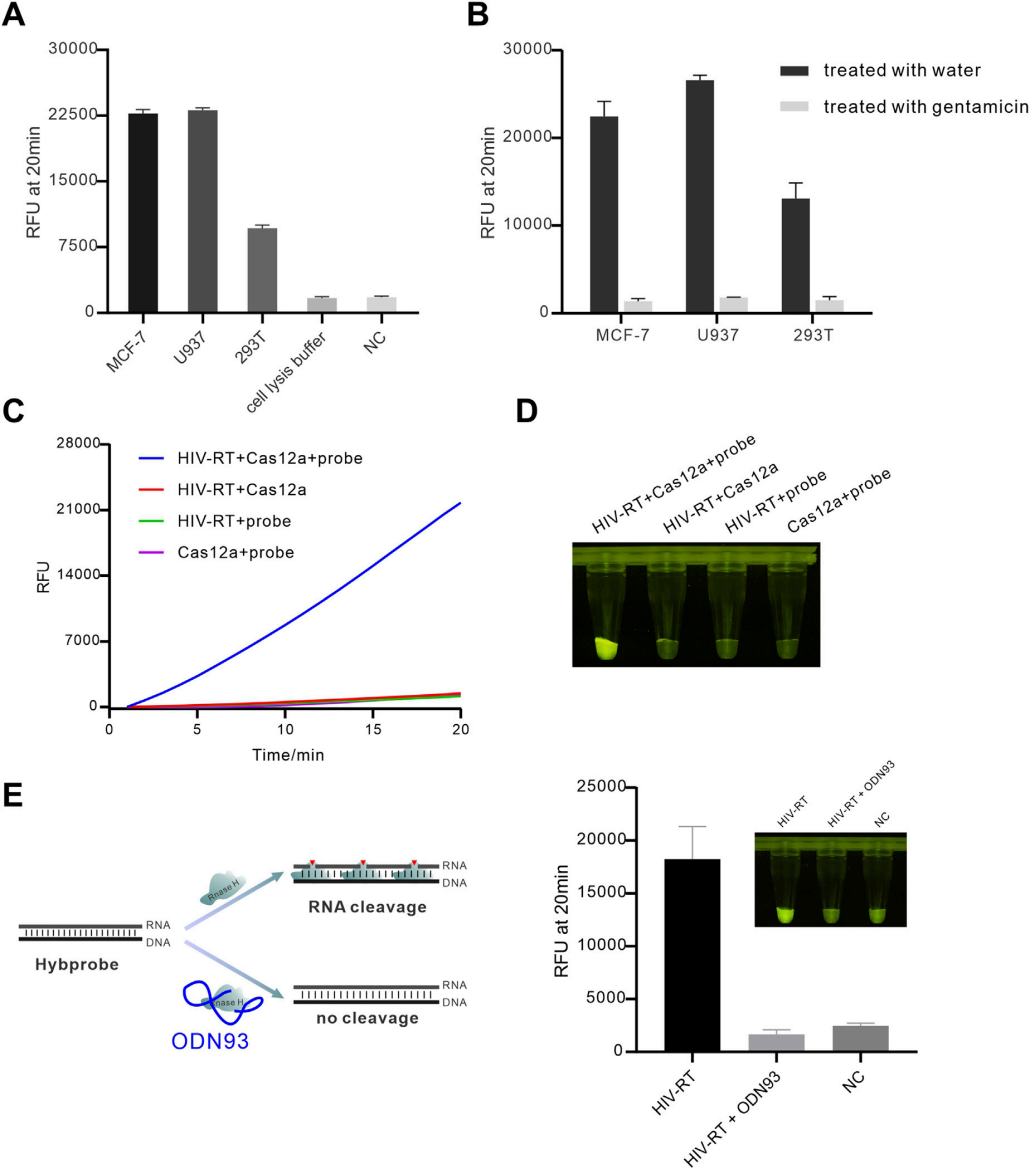
Detecting of cellular and viral RNase H. **(A)** Real-time fluorescence curves for different cell extracts were obtained; **(B)** investigation of the inhibition of cellular RNase H. The cell lysates were derived from approximately 50,000 cells, and the gentamicin concentration was 1 mM; **(C)** Real-time fluorescence curves for detection of RNase H activity of HIV reverse transcriptase; **(D)** Endpoint fluorescence image for detection of RNase H activity of HIV reverse transcriptase. The used concentration of HIV-RT was 31 nM. **(E)** Inhibition of HIV-RT RNase H activity by aptamer ODN93. The concentration of ODN93 was 1 uM.

To test the method’s applicability, we sought to applied the method to detect RNase H from different sources including human cell and retrovirus. In this study, we tested the cell extracts from different cell lines, including MCF-7, U937, and 293 T. The results showed that both three types of cell extracts induced strong fluorescent signals which could be distinctly discriminated from cell lysis buffer and negative control (Figure 6A). Interestingly, 293 T cell extract induced an obvious lower fluorescence intensity than other 2 cell extracts, which might be attributed to that MCF-7 and U937 were tumor cells that overexpress RNase H (Kimura et al., 2022). We also pretreated the cell extracts with gentamicin to evaluate the inhibition of cellular RNase H. A significant reduction in fluorescence signal was observed following gentamicin inhibition (Figure 6B), implying that our method could be used to screen the inhibitor targeting human cellular RNase H. Taken together, these results indicated that our method could be used to detect RNase H in human cells. Next, we attempted to investigate the viral RNase H activity. Acquired immunodeficiency syndrome (AIDS) is caused by the retrovirus known as HIV. Currently, many studies are focused on developing drugs targeting HIV replication-related proteins for the treatment of AIDS. RNase H plays an essential intermediate role in the replication of HIV, so developing RNase H inhibitor is of importance for the discovery of anti-HIV drugs (Tramontano, 2006). Since the RNase H active subunit of HIV is located within HIV reverse transcriptase, we evaluated our method using commercially available HIV reverse transcriptase (recombinant p66) in this study. As shown in Figures 6C, D, when HIV reverse transcriptase (HIV-RT) was added to the reaction, a strong fluorescence signal was observed, confirming that our method can be used for detecting RNase H activity in HIV. It was reported that a DNA aptamer, namely, ODN93, can inhibit the RNase H activity of HIV-RT (Andreola et al., 2001; Métifiot et al., 2005). Therefore, we used ODN93 as a model inhibitor to inhibit of HIV-RT. As shown in Figure 6E, we preincubated ODN93 with HIV-RT for 5 min and then added it to the detection reaction. The fluorescence intensity significantly decreased compared to the control group without ODN93, confirming that ODN93 could effectively inhibit the RNase H activity of HIV-RT. Additionally, we confirmed ODN93 did not affect the trans-cleavage activity of Cas12a (Supplementary Figure S6). Therefore, our method was capable of preforming inhibition analysis of HIV RNase H activity, showing great promise to screen potential antiviral prodrugs.

## Discussion

The CRISPR-Cas system, as a novel biosensing tool, seamlessly integrates target recognition with signal amplification, resulting in a highly adaptable biosensor. Its features, such as ease of use, precise detection, and high sensitivity, are exactly the core elements needed for the advancement of biosensing technology. After nearly 10 years of technological evolution, CRISPR/Cas-based detection platforms have been successfully applied to detect both nucleic acid and non-nucleic acid biomarkers, fully showcasing the wide applicability of this technology in the field of biosensing.

In this study, a hybrid probe coupled with Cas12a signal amplifier was proposed to develop a novel RNase H detection system. The method can detect RNase H with ultra-high sensitivity, down to 9.02 × 10^-10 U/μL. Additionally, by simultaneously generating crRNA and Hybprobe, the detection process can be performed easily and cost-effectively. This method has been successfully applied to the detection of RNase H activity from various sources, including human cells and HIV. In summary, the method has several advantages: (i) the entire detection process can be performed under constant temperature conditions, requiring fewer tools and being more cost-effective, with great potential for field detection applications; (ii) its high sensitivity enables both qualitative and quantitative detection of trace amounts of RNase H in various biological samples; (iii) the feasibility of inhibition assays provides new options for the development of prodrugs related to targeting RNase H. Therefore, this Hybprobe-based detection method provides a promising alternative for the detection of RNase H.

## Data Availability

The original contributions presented in the study are included in the article/Supplementary Material, further inquiries can be directed to the corresponding authors.
