# Supplementary material for: Ultrasensitive RNase H activity detection using the transcription-based hybrid probe and CRISPR/cas12a signal amplifier
Source: Front Pharmacol. 2025 Apr 11;16:1589150. doi: 10.3389/fphar.2025.1589150 (PMC12021801; doi:10.3389/fphar.2025.1589150)
Supplement: Supplementary file 1 [file DataSheet1.docx]

***Supporting materials***

**Ultrasensitive RNase H activity detection using the transcription-based hybrid probe and CRISPR/cas12a signal amplifier**

**Table S1. Sequence information used in this study**

| name | sequence(5′-3′) | role in the study |
| --- | --- | --- |
| T7F | GAAATTAATACGACTCACTATAGGG | HybProbe production |
| synthetic crRNA | AAUUUCUACUGUUGUAGAUUUGAACUGUUGCGACUACGU | Feasibility validation |
| ssDNA activator | ACGTAGTCGCAACAGTTCAAATCTACAACAGTAGAAATT | Feasibility validation |
| Single-stranded DNA reporter (ssRepoter) | FAM-TCCCCT-BHQ1^1^ | Cas12a signal amplification |
| HybProbe tamplate | ACGTAGTCGCAACAGTTCAAATCTACAACAGTAGAAATTCCCTATAGTGAGTCGTATTA | HybProbe production |
| ODN93 | GGGGGTGGGAGGAGGGTAGGCCTTAGGTTTCTGA | HIV RNase H inhibition |

The ssReporter was labeled with 5’ FAM (fluorophore) and 3’BHQ1 (quencher).

**Table S2. Comparison of the analytical performance of some reported methods in RNase H detection**

| Method | Detection technique | LOD | Operation complexity | time | temperature | Ref. |
| --- | --- | --- | --- | --- | --- | --- |
| end-point method based on graphene oxide | Fluorescence | 5.0×10^-3 U/ mL | high | 50 min | 37 ℃ | 1 |
| Tb3+-induced G- quadruplex conjugates | Fluorescence | 2 U/mL | high | 40 min | 37 ℃ | 2 |
| cascade primer exchange reaction with CRISPR/Cas12a system | Fluorescence | 6.1×10^-4 U/mL | moderate | >2 h | 37 ℃ | 3 |
| nicking enzyme-assisted fluorescence signal amplification based on a G-quadruplexe/thioflavin T complex | Fluorescence | 0.03 U/mL | high | 65 min | 37 ℃ | 4 |
| **Hybprobe H-coupled with CRISPR/Cas12a system** | **Fluorescence** | **9.02×10^-7 U/ mL** | **high** | **45 min** | **37 ℃** | **This work** |

**Table S3. The cost of the whole test**

| Content/reaction | Price/reaction | Total price/reaction |
| --- | --- | --- |
| Cas12a | $0.69 | $1.65 |
| T7 RNA transcriptase | $0.87 |  |
| T7F | $0.01 |  |
| ssRepoter | $0.06 |  |
| HybProbe tamplate | $0.02 |  |


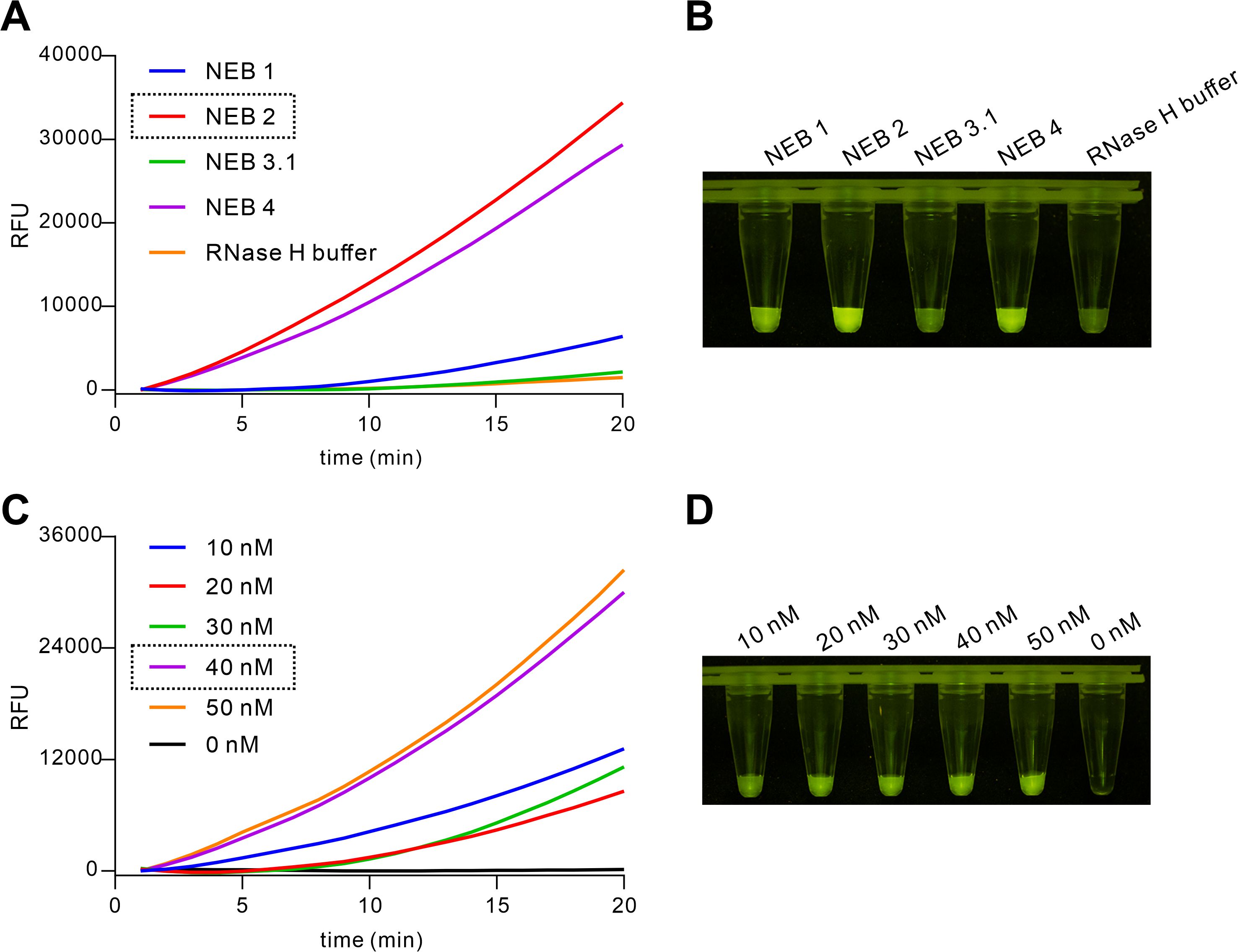


**Figure S1.** Optimization of reaction buffer and Cas12a concentration. (A) Examination of the detection buffer; (B) Endpoint visualization results of Figure A; (C) Optimization of Cas12a concentration; (D) Endpoint visualization results of Figure C. The RNase H concentration is 0.005 U/μL.


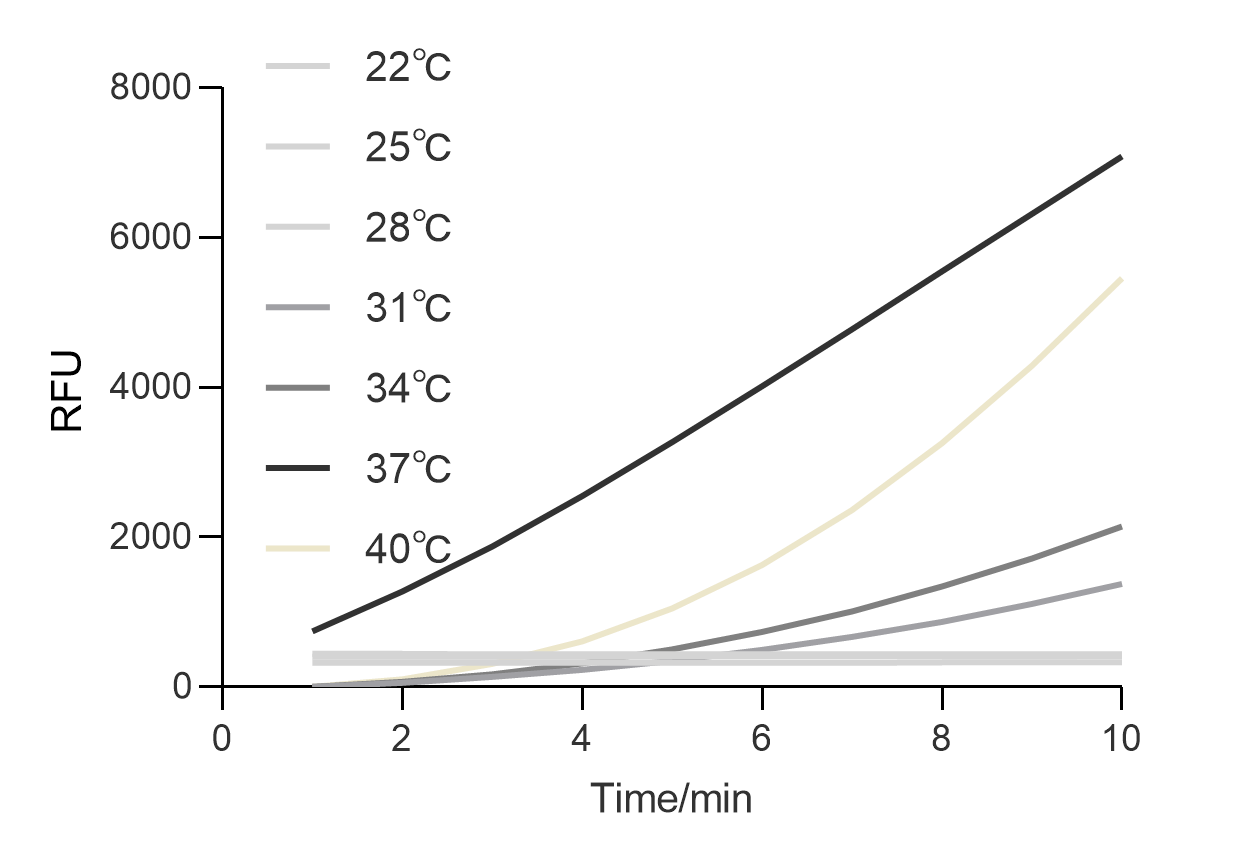


**Figure S2.** Optimization of reporting temperature. The RNase H concentration is 0.005 U/μL.


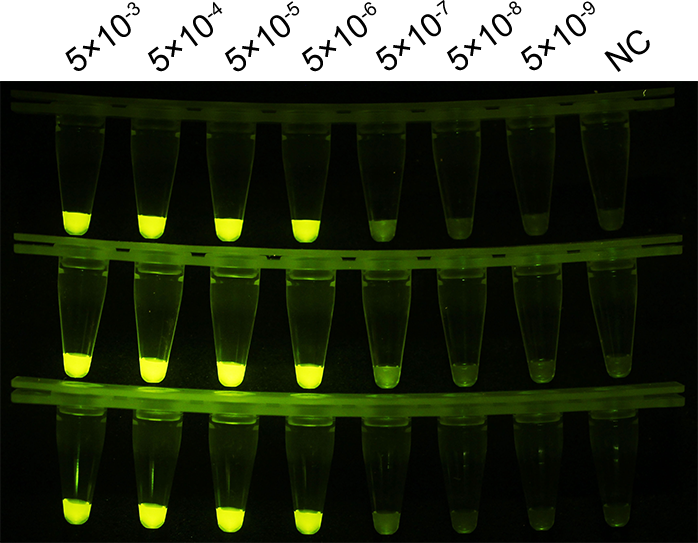


**Figure S3.** Endpoint fluorescence intensity at different RNase H concentrations.


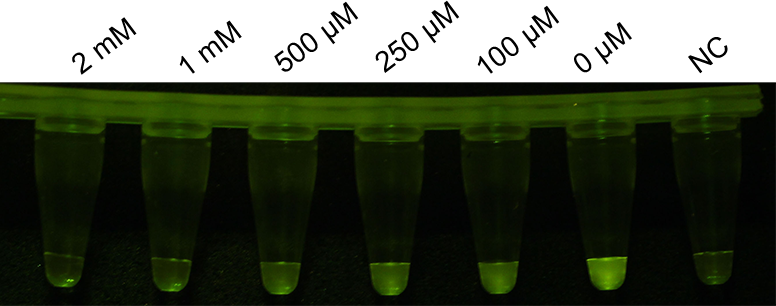


**Figure S4.** Endpoint fluorescence intensity at different gentamicin concentrations.


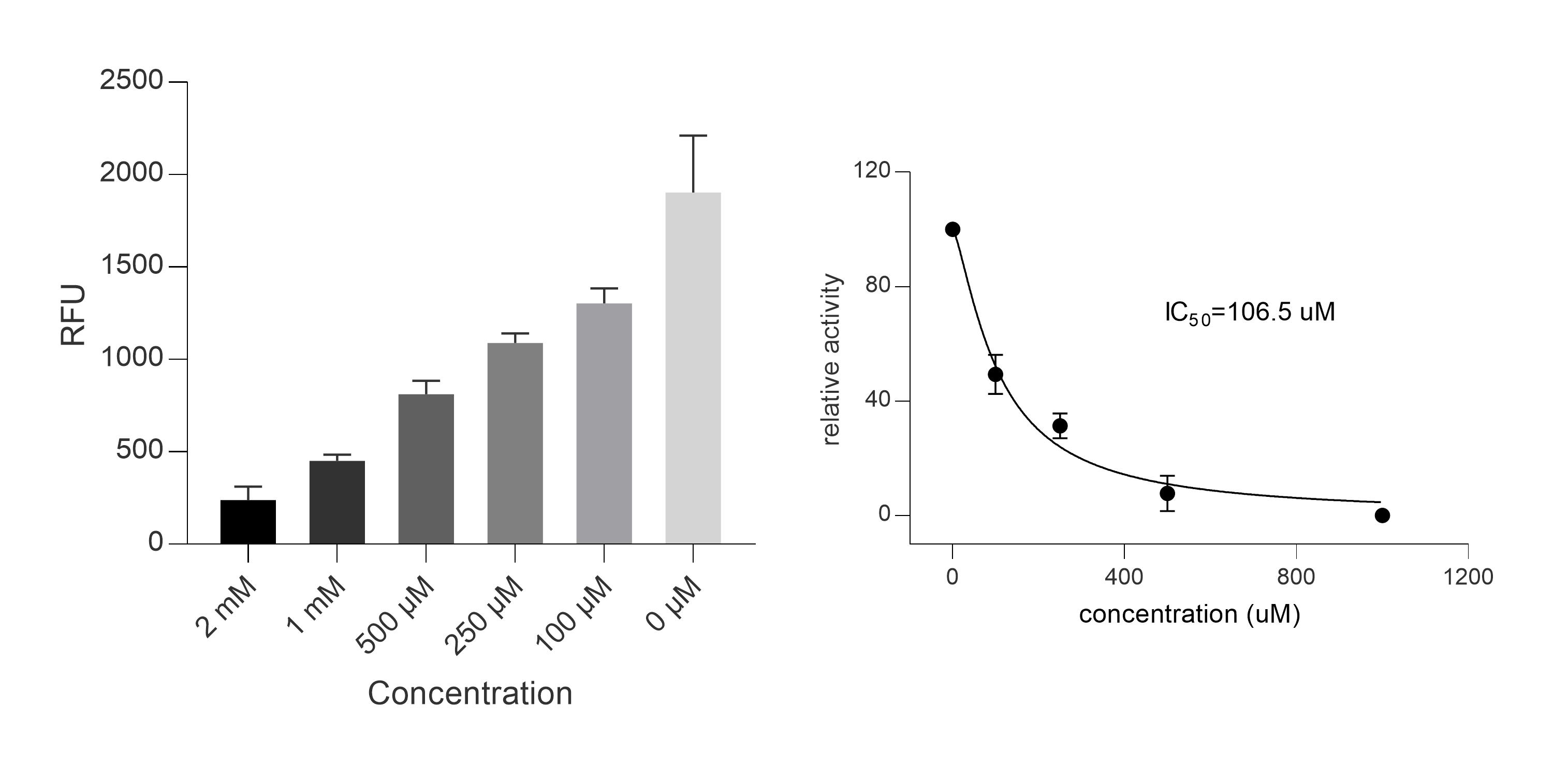


**Figure S5.** Inhibition assay of RNase H using streptomycin. The final concentration of RNase H was 1×10^-5 U/μL.


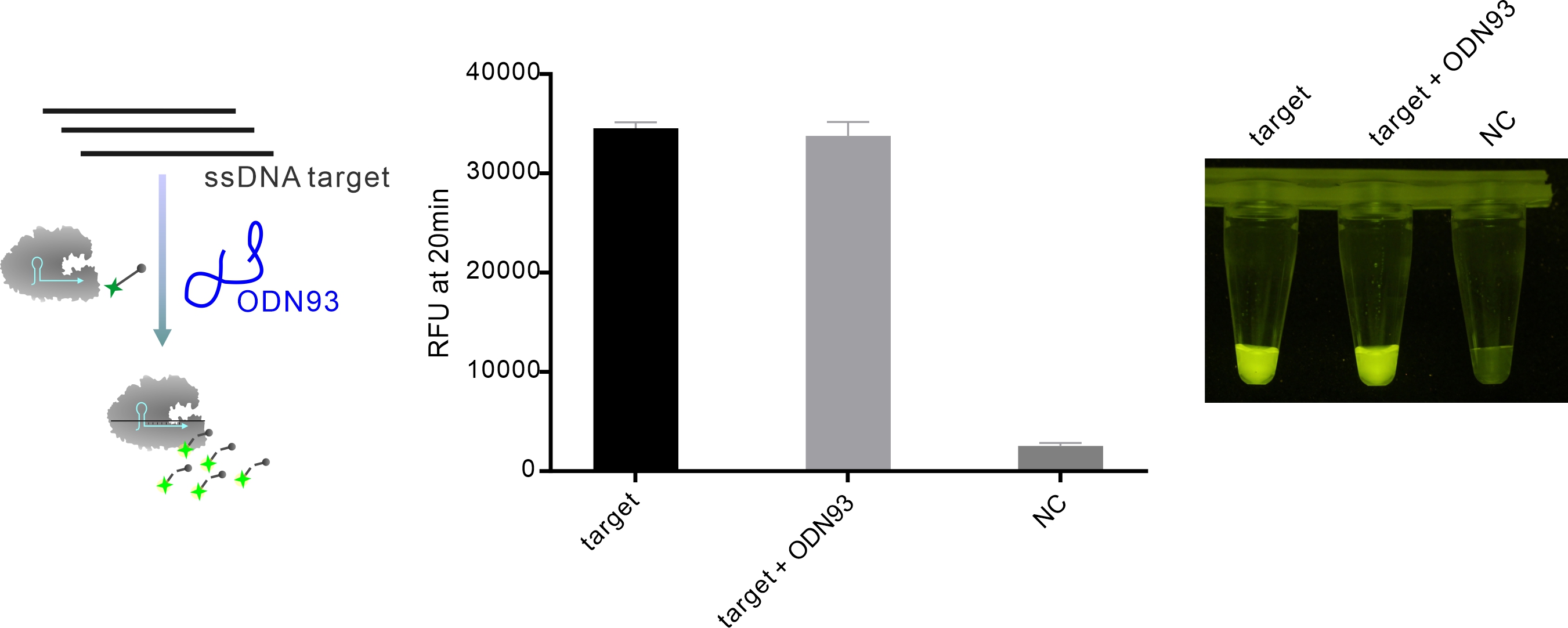


**Figure S6.** The effect of aptamer ODN93 on Cas12a trans-cleavage activity. The concentration of ODN93 was 1 μM, and the concentration of Cas12a was 40 nM.

**Reference:**

(1) Zhao, C.; Fan, J.; Peng, L.; Zhao, L.; Tong, C.; Wang, W.; Liu, B. An end-point method based on graphene oxide for RNase H analysis and inhibitors screening. *Biosensors and Bioelectronics* **2017**, *90*, 103-109. DOI: https://doi.org/10.1016/j.bios.2016.11.032.

(2) Wu, K.; Ma, C.; Liu, H.; He, H.; Zeng, W.; Wang, K. Label-free fluorescence assay for rapid detection of RNase H activity based on Tb3+-induced G-quadruplex conjugates. *Anal Methods-Uk* **2017**, *9* (20), 3055-3060, 10.1039/C7AY00709D. DOI: 10.1039/C7AY00709D.

(3) Xie, Z.; Chen, S.; Zhang, W.; Zhao, S.; Zhao, Z.; Wang, X.; Huang, Y.; Yi, G. A novel fluorescence amplification strategy combining cascade primer exchange reaction with CRISPR/Cas12a system for ultrasensitive detection of RNase H activity. *Biosensors and Bioelectronics* **2022**, *206*, 114135. DOI: https://doi.org/10.1016/j.bios.2022.114135.

(4) Wu, K.; Ma, C.; Deng, Z.; Fang, N.; Tang, Z.; Zhu, X.; Wang, K. Label-free and nicking enzyme-assisted fluorescence signal amplification for RNase H determination based on a G-quadruplexe/thioflavin T complex. *Talanta* **2018**, *182*, 142-147. DOI: https://doi.org/10.1016/j.talanta.2018.01.075.
